# Barriers of access and utilization of reproductive health services by adolescents-Khartoum state-Sudan-2020: study protocol

**DOI:** 10.1186/s12978-020-00967-y

**Published:** 2020-08-14

**Authors:** Mustafa Khidir Mustafa Elnimeiri, Shahenaz Seifaldeen Mustafa Satti, Mohanad Kamaleldin Mahmoud Ibrahim

**Affiliations:** 1 Preventive Medicine & Epidemiology, Alneelain University, Khartoum, Sudan; 2 Clinical Physiology, Faculty of Medicine, Alneelain University, Khartoum, Sudan; 3 Community Medicine & Epidemiology, Ibn Sina University, Faculty of Medicine, Khartoum, Sudan

**Keywords:** Adolescents:, Reproductive health,barrier of access

## Abstract

**Background:**

Adolescence is widely defined as the time in life when the developing individual attains the skills and attributes necessary to become a productive and reproductive adult. Most adolescents are healthy, but there is still substantial premature death, illness, and injury among adolescents. Illnesses can hinder their ability to grow and develop to their full potential. Alcohol or tobacco use, lack of physical activity, unprotected sex and/or exposure to violence can jeopardize not only their current health, but also their health as adults or even health of their future children.

**Method:**

Community and institutional-based cross sectional study will be conducted in Khartoum State the seven localities will be included. This state is the national capital of Sudan, which has an area of 22,122 km^2^. The sample size of participant is estimated using the population formula (n = N/1+ (n*d^2^)). The sample will be drawn using multistage cluster sampling. Also the concerned bodies involved in the delivery of reproductive health services for adolescents included in this study. Data will be collected using interviews with key informants and administered pre-coded, pretested closed ended questionnaire with community participants. Data will be managed and analyzed using Statistical Package for Social Sciences version 21. Analysis is mostly univariate descriptive and bi-variate with Chi Square & Fischer Exact test analysis to find associations between variables of interest.

**Discussion:**

The census of adolescents mounted to 25% of the population and thus it is important to care for such significant portion of the population to document their reproductive health problems and their access to health care services. The study is expected to generate base-line indicators about barriers of access to reproductive health services by adolescents that can be used for better planning, monitoring and evaluation of the delivered services. The research about barriers of access to reproductive health services by adolescents in Sudan is still limited and the information is scanty and scattered. Thus, it is necessary to conduct such study to enrich the current database.

## Plain English summary

Adolescents (10–19 years) are the future leaders of the societies, they are perceived to be healthy, and however, they are vulnerable and exposed to many health risks leading to premature deaths. Adolescent sexual and reproductive health access must dominate the health system stratagems in the developing countries. The barrier for access to reproductive health range from facility level barrier, social barrier, provider level barrier. Khartoum state is the Capital of Sudan, according to 2008 population census/projections 2018; the population of Khartoum State is estimated to be 7,993,852 people, who are a mixture of different Sudanese tribes and ethnic groups,

The adolescent population in Khartoum State, projection 2018, the male adolescents mounted to 923,735 (52.6%) while the female adolescents mounted to 832, 746 (47.4%). The demographic structure of the adolescent sector was almost similar to the parent population of the state, and composed of different Sudanese tribes and ethnic groups. This study protocol is designed to verify the b*arriers of access and utilization of reproductive* health services, to identify the major reproductive health problems, health care seeking practices of the adolescents in Khartoum state aiming to avail information for better interventions to improve the health care service.

## Background

Adolescence is widely defined as the time in life when the developing individual attains the skills and attributes necessary to become a productive and reproductive adult. Nearly all cultures recognize a phase in life when society acknowledges these emerging capacities of young people. What varies considerably by culture and context is whether the passage from childhood to adulthood is a direct and the short passage, or whether there is a prolonged adolescence marked by a choice of identities and roles [1].

There are nearly about 1.2 billion adolescents worldwide while in some countries they compose around 25% of the total population. The census is expected to rise by year 2050 especially in low and middle-income countries where 90% of adolescents live [2]. This has marked implications on the commitment of the governments in these countries towards achieving universal health coverage (UHC), a core component of the sustainable development goals (SDGs) by the year 2030 [2].

Most adolescents are healthy, but there is still substantial premature death, illness, and injury among adolescents. Illnesses can hinder their ability to grow and develop to their full potential. Alcohol or tobacco use, lack of physical activity, unprotected sex and/or exposure to violence can jeopardize not only their current health, but also their health as adults, and even the health of their future children [3]..

During adolescence, an individual acquires the physical, cognitive, emotional and social resources that are the foundation for later-life health and well-being. These resources define trajectories into the next generation [4]. The health attitudes, attributes and behaviors that are developed and cemented during adolescence, which begins at age 10, will define a girl’s health outcomes throughout her life. Positive choices during this critical period in a girl’s life, and access to youth-friendly health services have lifelong effects [5].

Global interest in the health of adolescents and youth has manifested itself in the many expressions of commitment to their healthy personal, spiritual, social, mental and physical development. The 1990s saw the affirmation of worldwide commitments to adolescent and youth health that have been shaped within an international legal framework that has as its foundation the United Nations Charter [6] and that reflects the WHO definition of health as a state of complete physical, mental and social wellbeing and not merely the absence of disease or infirmity [7]. One implication is that the international public health community must adopt an approach to adolescents and youth that goes beyond the health sector to elicit the active participation of all social actors, including young people themselves as agents of change [8].

The services, commodities, information and skills needed to sustain healthy behavior must be provided in the safest and most supportive of environments, building on the protective factors of family and community [9]*.*

**Table:** 

OBJECTIVES	
This research is designed to study the b*arriers of access and utilization of reproductive* health services by adolescents aiming to avail information for better interventions Specific objectives: 1. To identify the major reproductive health problems of adolescents 2. To identify the reproductive health care seeking practices of the adolescents 3. To verify the barriers of access and utilization of reproductive health care services by the adolescents	

## Methodology

A community and institutional-based cross sectional based study will be conducted in Khartoum State capital of Sudan, which is one of the 18 states of Sudan. It has an area of 22,122 km^2^. Khartoum State is subdivided administratively into seven localities namely; Khartoum, Khartoum North, Omdurman, Jabal Awliya, Sharq an-Nīl, Ombadda and Karari Localities. Each locality is subdivided into administrative units and each administrative unit is subdivided into local committees [10]. According to 2008 population census/projections 2018, the population of Khartoum State is estimated to be 7,993,852 people who are a mixture of different Sudanese tribes and ethnic groups. As to the activity of the population of Khartoum State, it can be said that most of the population are workers and personnel in the State chambers, the private sector and banks. Also, there is a large segment of capitalists dealing in trade and another segment represented by migrants and displaced people working in marginal activities. As to the countrymen, they are engaged in agriculture, grazing and thus supply the capital, Khartoum, with vegetables, fruits, dairy. There are also some residents who live on the banks of the river engaged in the river-related work such as pottery, brick and fishing [10].

### The adolescent population

The total census of the adolescent population in Khartoum State, projection 2018 [11]. The male adolescents mounted to 923,735 (52.6%) while the female adolescents mounted to 832, 746 (47.4%). The demographic structure of the adolescent sector was almost similar to the parent population of the state, and composed of different Sudanese tribes and ethnic groups. We include in the study the Sudanese10–19 years of age, Both gender,Mentally healthy .

### The policy and decision makers (key informants) involved in the delivery of reproductive health services to adolescents

The population is composed of concerned bodies involved in the delivery of reproductive health services for adolescents including policy-makers, decision makers at the Federal Ministry of Health, Khartoum State Ministry of Health, Social affairs, National Council of Child welfare, UN agencies, NGOs, civil society organizations and health care providers.

### Sample size

The sample size of the adolescent population will be estimated using the following formula [12].

$$ \mathrm{n}=\mathrm{N}/1+\Big(\mathrm{N}\ast {\mathrm{D}}^{2\Big)} $$

Where:

N: sample size.

N: Total population = 1, 756,481

E: degree of precision = 0.05

Thus the estimated sample size (n) = 399.9 ≈ 400

Since the sampling technique planned to be used is multi-stage sampling technique rather than simple random technique; then it is necessary to multiply the estimated sample size by the design effect, which is approximately equal to 2 in order to improve representation.

Thus the sample size = 400X2 = 800

On the assumption that the non-response rate is 15%, then the final estimated sample size = 920.

Thus the final sample is composed of both male and female adolescents (proportional to size) as follows:

Male adolescents: 483

Female adolescents: 437

### Sampling method

A multi-stage cluster sampling technique will be used to draw the sample. The sample will be drawn through the following steps:

- Step (1): The study will be conducted in 50% of the localities, which will be selected using simple random sampling technique.
- Step (2): The administrative units within the selected localities are be subdivided into clusters based on the geographical locations. Then 50% of the administrative units within the selected localities will be selected using simple random sampling technique.
- Step (3): The committees within the selected administrative units is subdivided into clusters based on the geographical locations. Then 50% of the committees within the selected administrative units are selected using simple random sampling.
- Step (4): Each of the selected committees is subdivided into clusters of households and then 50% of the clusters are selected. The estimated sample size is distributed proportional to size of the population and gender census in each of the selected committees.
- Step (5): The households within the selected clusters are identified and then the adolescents.

Within the households are interviewed.

As regards the sample of the concerned bodies in organizations involved in delivery of reproductive health services for adolescents, total coverage of the policy makers, decision makers and managers will be used. The number of the key informants is estimated to be around 25.

### Data collection instruments

A standardized administered questionnaire will be developed, pre-tested and used for quantitative data collection from interviewed adolescents (A questionnaire for each gender). The questionnaire of the female adolescents will be composed of about 42 close-ended questions while the questionnaire of the male adolescents will be composed of 32 close-ended questions. The questionnaire is composed of the following chapters:

- Chapter (1): Personal & family characteristics: age, educational level, monthly income, family type.
- Chapter (2): Reproductive health problems & services: RH problems i.e. FGC complications, sexually transmitted infections, menstrual disorders among the adolescents and treatment seeking and place of seeking treatment.
- Chapter (3): Barriers of access and utilization of RH services: personal, structural, socio-cultural barriers.

Focus group discussion guidance: Ten structured focus group discussions, five per gender, will be conducted to generate qualitative data. A structured guidance for the focus group discussion will be prepared and used for collection of data by the trained data collectors. The guidance will be composed of seven open-ended and focused on variables not addressed in the questionnaire.

An interview guidance will be developed, pre-tested and used for qualitative data collection from key informants in the concerned bodies involved in delivery of RH services for adolescents. The guidance will be composed of 13 open-ended questions covering policy, strategic barriers and gender issues relevant to delivery of RH services for adolescents.

### The pre-testing of the data collection instruments

All the data collection instruments will be developed by the principle investigator and then experts in the various disciplines will revise the instruments in order to ensure internal validity. Then the questionnaire will be pre-tested through administration to six male and female adolescents in a locality other than the localities included in the sample. Necessary corrections and adjustments will be carried out based on the reported results of the pre-testing.

The data collection team will be composed of 4 field supervisors and 20 data collectors of both genders. The data collectors will be of close age to the study subjects as this is important in dealing with interviewed adolescents [13]. In addition, they will be interested and motivated to work with full respect of dignity of the study participants and confidentiality required during data collection process.

#### Data management plan

The data collectors in the field will employ portable handheld computers (tablet PC) for data collection; the data manager will design a data entry template using the Statistical Package for Social Sciences (SPSS) version 23.

Then the data manager who is a qualified statistician with adequate knowledge and skills in using SPSS performs data cleaning and ensures correctness and consistency of responses.

Analysis is mostly univariate descriptive in order to generate indicators of interest. In addition, bi-variate analysis will be conducted to find associations between variables of interest. Since most of the variables are categorical, associations between variables are determined using non-parametric tests such as Chi Square & Fischer Exact tests. Analysis outputs will be displayed as tabular and graphic formats. The collected qualitative data will be validated and content analysis will be held.

## Discussion

This research is designed to explore the major reproductive health problems of adolescents and to verify the barriers of access and utilization of reproductive health care services by the adolescents.

The health systems in most of the countries do not adequately address the needs of the adolescents at all or inadequately responsive to such needs [2]. Other studies indicated that the reproductive health services for adolescents are inadequate with poor utilization and accessibility [14, 15]. The barriers to adolescent access to reproductive health services were categorized as individual, socio-cultural, structural and policy barriers [16].

A comprehensive analysis of population health data by Patton et al. clearly shows that young people are at substantial risk of mortality, with a pronounced rise in mortality rates from early adolescence (10–14 years) to young adulthood (20–24 years) and higher concentration in developing countries [17].

Another study in South Africa revealed the barriers of access to reproductive health as lack of confidentiality and privacy, waiting time duration, unpleasant work hours, distance from clinics, and parents’ fear when they came to the clinic [18]. Secor-Turner MA & et al. reported social stigma, fear of non-confidentiality of services, and the prohibition of their parents and tutors, teenagers as the main barriers of adolescents’ access to barriers [19].

The Multiple Indicators Survey Adolescents Health survey in Khartoum State [20] revealed that 79.3% of male adolescents and 79.5% of female adolescents respectively were seeking treatment from governmental hospitals/centers if felt sick. 81% of male adolescents sought that the provided services fulfilled their needs as adolescents while 71% of female adolescents sought that the provided services fulfilled their needs as adolescents. The reasons for not fulfilling the adolescents’ needs were mainly poor quality of the provided services and inadequate response to health services to their needs as adolescents.

This study will estimate Personal barriers encountered with access of reproductive health services by adolescents i.e. attitudes towards the RH services, fear of stigma, financial barriers,Socio-cultural barriers of access of reproductive health services for adolescents i.e. community stigma, community taboos, peer pressureand Structural barriers of access of reproductive health services by adolescents i.e. health providers’ attitude, availability of RH services, quality of RH services, geographical accessibility.

Policy barriers of access of reproductive health services by adolescents, i.e. gender issues, poor addressing of adolescents’ rights, poor coordination between concerned bodies.

## Data Availability

No data were generated during the current status of the study. Once the study is finalized and the results are published, a specific procedure for obtaining access to the database will be made.

## Abbreviations

UHC: Universal health coverage
WHO: world health organization
UN: united nation
RH: reproductive health
FGC: female genital cutting
PC: portable computer
SPSS: Statistical packages of social sciences
